# Coronectomy Versus Total Extraction for Third Molar Surgery: A Systematic Review and Meta-Analysis

**DOI:** 10.7759/cureus.105646

**Published:** 2026-03-22

**Authors:** Ali A Derbishi, Raand A Altayyar, Abdulaziz S Alsubaiei, Razan J Alghannam, Ahlam Albalushi, Ola Mubarki, Alanoud Arim, Ghusun F Alahmadi, Ayman Alothman, Arwa M Manaa, Rawabi A Almohaimeed, Renad A Alsum, Meaad B Howsawi, Ali S Metwaly

**Affiliations:** 1 Oral and Maxillofacial Surgery, Sabya General Hospital, Jazan, SAU; 2 Dentistry, College of Dentistry, Imam Abdulrahman Bin Faisal University, Dammam, SAU; 3 General Dentistry, Private Clinic, Muscat, OMN; 4 Dentistry, College of Dentistry, Jazan University, Jazan, SAU; 5 Dentistry, College of Dentistry, King Khalid University, Abha, SAU; 6 Oral and Maxillofacial Surgery, King Saud Mesical City, Riyadh, SAU; 7 Dentistry, Princess Nourah Bint Abdulrahman University, Riyadh, SAU; 8 General Dentistry, Faculty of Dentistry, King Abdulaziz University, Jeddah, SAU; 9 Precision Medicine, Faculty of Pharmacy, Alexandria University, Alexandria, EGY

**Keywords:** coronectomy, inferior alveolar nerve injury, meta-analysis, oral surgery, third molar surgery, tooth extraction

## Abstract

Surgical extraction of impacted mandibular third molars near the inferior alveolar nerve (IAN) carries a risk of neurosensory injury. Coronectomy (intentional partial odontectomy) is a neuroprotective alternative, but concerns regarding long-term complications like root migration and the necessity for re-operation persist. This systematic review and meta-analysis evaluates the comparative safety and efficacy of coronectomy versus total surgical extraction. A literature search was conducted across PubMed, Embase, Scopus, and Cochrane Library for randomized controlled trials (RCTs) and prospective cohort studies comparing coronectomy to total extraction in high-risk lower third molars. Primary outcomes included IAN injury, dry socket, and infection. Secondary outcomes included pain, root migration, and re-operation rates. Data were pooled using random-effects models (REML) with Hartung-Knapp-Sidik-Jonkman adjustment. Trial sequential analysis (TSA) was performed for the primary outcome. The certainty of evidence was assessed using Grading of Recommendations Assessment, Development, and Evaluation (GRADE). Eight studies (three RCTs, five cohorts) comprising 1,488 teeth were included. Coronectomy significantly reduced the risk of IAN injury compared to total extraction (Peto OR 0.23, 95% CI (0.13, 0.39), *p* < 0.0001). TSA confirmed that the evidence for IAN injury prevention is conclusive. No statistically significant differences were found between groups for dry socket (RR 0.68, *p* = 0.22) or postoperative infection (RR 0.87, *p* = 0.71). Root migration was a common physiological sequela, but the pooled rate of re-operation to retrieve roots was low at 1.2% (95% CI (0.0%, 4.4%)). Coronectomy is a superior neuroprotective technique for high-risk mandibular third molars, significantly reducing nerve injury without increasing the risk of infection or dry socket compared to total extraction. While root migration is frequent, secondary surgical intervention is rarely required. Coronectomy is a highly effective surgical alternative that should be prioritized for radiographically high-risk impactions.

## Introduction and background

The surgical extraction of impacted mandibular third molars is one of the most frequently performed procedures in oral and maxillofacial surgery [1]. Intervention is indicated for the management or prevention of various pathologic conditions, including pericoronitis, dental caries, periodontitis, cystic lesions, odontogenic tumors, and orthodontic complications [1,2]. While total surgical extraction is the gold standard for managing symptomatic third molars, the procedure is associated with a spectrum of postoperative complications, such as pain, swelling, trismus, dry socket (alveolar osteitis), and infection [3,4].

Among these complications, iatrogenic injury to the inferior alveolar nerve (IAN) is the most feared and clinically debilitating risk [5]. Neurosensory deficits of the IAN can manifest as temporary or permanent hypoaesthesia, paraesthesia, or dysaesthesia of the lower lip, chin, and gingiva, compromising the patient's quality of life [3]. The incidence of temporary IAN injury following conventional third molar extraction ranges from 0.5% to 8%, with permanent nerve damage occurring in approximately 1% to 4% of cases [3,4]. However, in high-risk cases where the tooth roots are in intimate anatomical proximity to the mandibular canal, the incidence of nerve injury can surge to nearly 36% [2]. Preoperative radiographic assessments, utilizing panoramic radiography to identify risk markers such as root darkening, canal diversion, or interruption of the canal's cortical white lines, often supplemented by three-dimensional cone-beam computed tomography (CBCT), are critical for identifying these high-risk impactions [5,6].

To mitigate the risk of neurologic morbidity in high-risk cases, coronectomy, or intentional partial odontectomy, has emerged as a viable surgical alternative [5,7]. First described in 1984 by Ecuyer and Debien, the procedure involves the surgical amputation and removal of the tooth crown while retaining the vital root complex within the socket [2,3]. By sectioning the tooth at least 3 mm below the buccal and lingual alveolar crests and achieving primary mucosal closure, the surgeon addresses the crown-related pathosis (e.g., pericoronitis) without exposing the adjacent IAN to the mechanical trauma of root elevation [5].

Despite its anatomical rationale and strong evidence suggesting a reduction in IAN injury rates, with some studies indicating up to an 84% reduction in risk compared to total extraction [3], coronectomy remains a subject of clinical debate. Critics of the technique highlight the potential for unique postoperative sequelae, including the coronal migration of the retained roots (reported in 2% to 85% of cases), failure of soft tissue closure leading to root exposure, and deep-seated infections of the root remnant [5,7]. When these complications occur, a secondary surgical intervention is often required to retrieve the retained roots, thereby incurring additional costs and patient morbidity [3,5]. Also, there is conflicting data regarding whether coronectomy influences the rates of common postoperative morbidities, such as dry socket, generalized pain, and periodontal healing of the adjacent second molar, compared to total extraction [4,7].

Because of the controversies surrounding the long-term fate of retained roots and the necessity for reoperation, a comprehensive and updated synthesis of the evidence is imperative. Therefore, the objective of this systematic review and meta-analysis is to evaluate the comparative efficacy and safety of coronectomy versus total surgical extraction for high-risk mandibular third molars. By analysing patient-reported outcomes, IAN injury rates, and long-term postoperative complications, this study aims to provide evidence-based guidance for clinical decision-making in third molar surgery.

## Review

Methods

Protocol and Registration

This systematic review and meta-analysis was conducted in adherence to the Preferred Reporting Items for Systematic Reviews and Meta-Analyses (PRISMA) guidelines [8]. The study protocol was prospectively registered in the PROSPERO international prospective register of systematic reviews (Registration Number: CRD420261305351).

Search Strategy and Eligibility Criteria

A systematic literature search was executed across electronic databases (e.g., PubMed/MEDLINE, Embase, Scopus, and the Cochrane Library) from inception to the present. The search strategy utilized a combination of Medical Subject Headings (MeSH) and free-text terms, including "coronectomy", "partial odontectomy", "third molar", "inferior alveolar nerve", and "extraction". Inclusion criteria were formulated based on the PICOS framework: (P) patients requiring the surgical management of mandibular third molars with a radiographically confirmed high risk of inferior alveolar nerve (IAN) injury, defined by the presence of at least one of the following signs on panoramic or CBCT imaging: darkening of the roots, diversion of the IAN canal, or interruption of the canal’s cortical white line; (I) coronectomy or intentional partial odontectomy; (C) total surgical extraction; (O) primary outcomes including IAN injury (temporary or permanent), and secondary outcomes including dry socket, infection, pain severity, root migration, and re-operation rates; and (S) randomized controlled trials (RCTs) and non-randomized clinical trials/prospective cohort studies. Case reports, reviews, and in vitro studies were excluded.

Study Selection and Data Extraction

Two independent reviewers screened the retrieved literature by titles and abstracts, followed by full-text evaluations to determine final eligibility. Discrepancies were resolved through arbitration by a third reviewer. The inter-rater reliability for study selection and data extraction was quantified using Cohen’s Kappa (κ) statistic to ensure reproducibility and mitigate selection bias [9].

Critical Appraisal and Risk of Bias

The methodological quality and risk of bias of the included studies were assessed using tool-specific paradigms. For randomized controlled trials, the Cochrane Risk of Bias 2.0 (RoB 2) tool was employed to evaluate domains such as the randomization process, deviations from intended interventions, missing outcome data, measurement of the outcome, and selection of the reported results [10]. For non-randomized interventional studies and prospective cohorts, the Risk of Bias in Non-randomized Studies - of Interventions (ROBINS-I) tool was utilized to assess confounding, selection, classification, and reporting biases [11].

Effect Synthesis and Statistical Modelling

Treatment effects for dichotomous clinical outcomes (e.g., dry socket, postoperative infection) were expressed as Odds Ratios (OR) and Risk Ratios (RR) [12]. Given that IAN injury is a rare adverse event, standard pooling can introduce statistical artifacts; thus, the Peto Odds Ratio method was utilized specifically for pooling rare events and handling studies with zero-cell counts without artificial continuity corrections [13]. Continuous outcomes (e.g., pain visual analog scale (VAS) scores, root migration distance) were pooled using Mean Differences (MD) or Standardized Mean Differences (SMD) based on the uniformity of the measurement scales [12].

A Random-Effects Model, incorporating both Restricted Maximum Likelihood (REML) and DerSimonian-Laird variance estimators, was established a priori to account for anticipated clinical and methodological heterogeneity across surgical protocols and patient demographics [14]. To counteract the inflated false-positive rates typical in meta-analyses with a limited number of studies, the Hartung-Knapp-Sidik-Jonkman (HKSJ) adjustment was applied, yielding highly robust and conservative 95% Confidence Intervals (CIs) [15]. Furthermore, 95% Prediction Intervals were calculated to estimate the range in which the true treatment effect will fall for a future clinical setting, enhancing the translational value of the findings [16].

For single-arm proportional outcomes unique to the coronectomy cohort (e.g., overall incidence of root migration, root exposure, and the need for re-operation), event rates were stabilized using the Freeman-Tukey double arcsine data transformation before pooling [17]. To facilitate clinical decision-making, pooled relative risk metrics were converted into absolute risk measures, specifically the Number Needed to Treat (NNT) to prevent one IAN injury, and the Number Needed to Harm (NNH) for associated adverse events [18].

Heterogeneity, Variability, and Robustness

Statistical heterogeneity (inconsistency) was evaluated using Cochran’s Q test (significance defined as P < 0.10) and quantified using the I2 statistic, representing the percentage of total variation across studies attributable to heterogeneity rather than chance. The absolute dispersion of true effect sizes was quantified using τ2 (Tau-squared) [19].

To explore sources of heterogeneity, moderation analyses via subgroup stratifications (e.g., RCT vs. non-RCT, imaging modality (CBCT vs. panoramic radiography), follow-up duration) and meta-regression models were executed [20]. The robustness of the summary estimates was interrogated using sensitivity analyses, specifically a leave-one-out paradigm, to ascertain if any single study disproportionately influenced the pooled effect [20].

Publication Bias and Small-Study Effects

Potential reporting and dissemination biases were evaluated through the visual assessment of funnel plots for asymmetry. When at least 10 studies were available for a given outcome, small-study effects were formally tested using Egger’s linear regression test [21] and Begg’s rank correlation test [22].

Trial Sequential Analysis (TSA)

To overcome the limitations of traditional meta-analyses regarding random errors and repetitive testing, a Trial Sequential Analysis (TSA) was performed. TSA calculated the required information size (optimal sample size) and established trial sequential monitoring boundaries. This post hoc assessment determined whether the cumulative evidence for critical outcomes (e.g., IAN injury prevention) was statistically conclusive or if further trials are warranted [23].

Overall Evidence Grading

The certainty and overall strength of the evidence for each critical outcome were appraised using the Grading of Recommendations Assessment, Development, and Evaluation (GRADE) framework. Evidence was adjudicated as high, moderate, low, or very low quality based on risk of bias, inconsistency, indirectness, imprecision, and publication bias [24].

Software

All statistical syntheses, meta-regressions, and plot generations were performed using R statistical software version 4.5.2 (R Foundation for Statistical Computing, Vienna, Austria) [25], utilizing the meta, metafor, and dmetar packages.

Results

Study Selection and Characteristics

**Figure 1:**
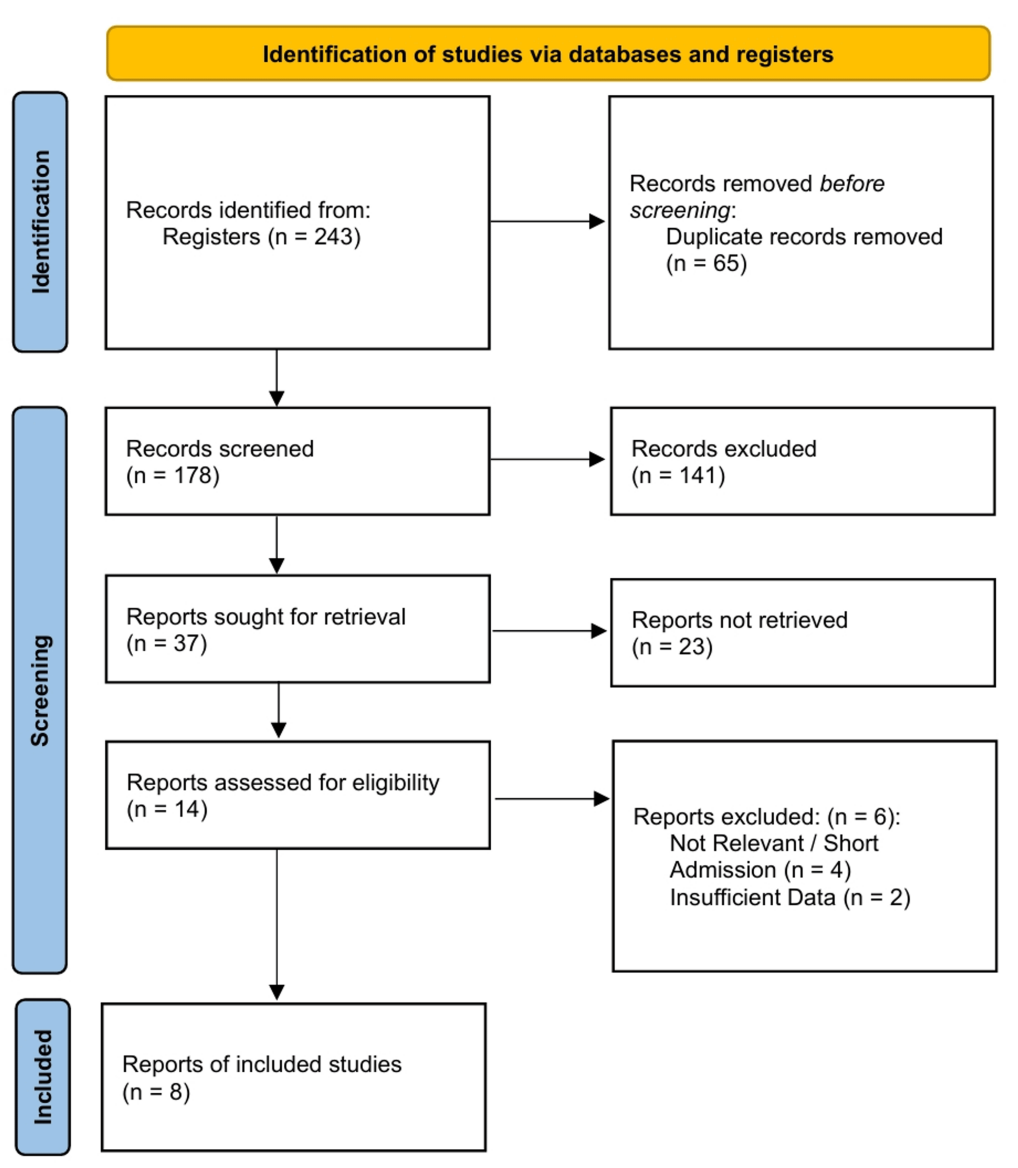
PRISMA Flow Diagram. PRISMA: Preferred Reporting Items for Systematic Reviews and Meta-Analyses

The systematic literature search and study selection process were conducted in accordance with Preferred Reporting Items for Systematic Reviews and Meta-Analyses (PRISMA) guidelines (Figure 1). Following abstract and full-text screening, a total of eight studies (three RCTs and five Non-Randomized Clinical Trials/Prospective Cohorts) met the inclusion criteria (Table 1) [26-33]. The inter-rater reliability for study selection between the independent reviewers demonstrated substantial agreement (Cohen’s κ = 0.69).

**Table 1:** Characteristics of Included Studies. CBCT, Cone-Beam Computed Tomography; Coro, Coronectomy group; Ext, Total Extraction group; IANI, Inferior Alveolar Nerve Injury; PoSSe, Postoperative Symptom Severity scale; QST, Quantitative Sensory Testing; RCT, Randomized Controlled Trial.

Study ID	Design	Country	Imaging Modality	Total Teeth (Patients) Coro / Ext	Mean Age (Years) Coro / Ext	Max Follow-up	Primary Outcomes Assessed
RCTs							
Leung & Cheung 2009 [28]	RCT	China	Panoramic	171 / 178	27.2 / 26.2	24 Months	IANI, Dry Socket, Infection, Migration
Pang et al. 2024 [29]	RCT (Split-mouth)	Hong Kong	CBCT	40 / 40	26.7 (Overall)	6 Months	Periodontal Healing, IANI
Renton et al. 2005 [30]	RCT	UK	Panoramic	94 / 102	29.0 / 27.5	25 Months	IANI, Dry Socket, Infection
Non-RCTs							
Al-Ali et al. 2025 [26]	Cross-Sectional	UAE	Panoramic	35 / 35	31.0 (Overall)	2 Weeks	Patient-Reported Outcomes (PoSSe), Pain
Cilasun et al. 2011 [33]	Prospective Cohort	Turkey	CBCT	88 / 87	27.2 / 27.4	30 Months	IANI, Dry Socket, Failed Coronectomy
Hamad 2024 [27]	Prospective Cohort	Iraq	CBCT	220 / 218	31.7 / 20.9	24 Months	IANI, Dry Socket, Root Migration
Kang et al. 2019 [32]	Prospective Cohort	China	CBCT	55 / 55	26.5 / 25.3	36 Months	IANI, Long-term Migration, Inflammation
Yan et al. 2020 [31]	Prospective Cohort	China	CBCT	91 / 49	27.2 / 28.0	6 Months	Somatosensory Function (QST), IANI

Risk of Bias Assessment

**Figure 2:**
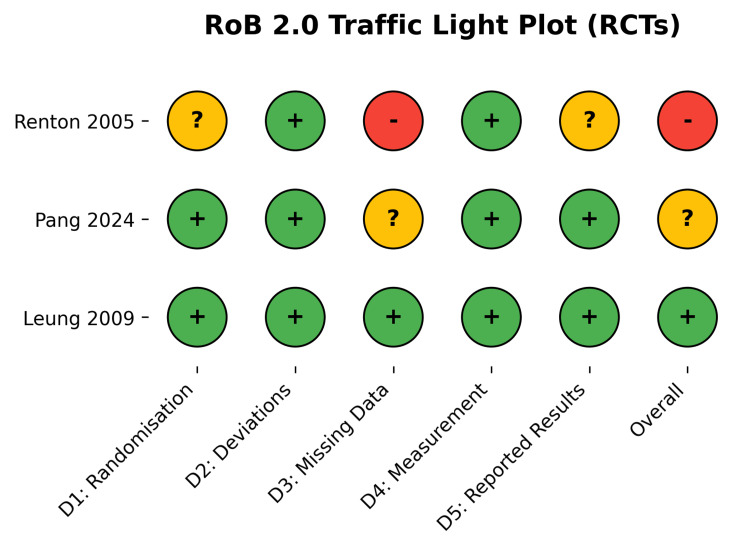
RoB 2.0 Traffic Light Plot Detailing Domain-Specific Bias for Included RCTs. The colors and symbols represent the domain-specific and overall risk-of-bias judgments according to the Cochrane RoB 2.0 and ROBINS-I tools. Green circle with a '+' (plus) symbol: Low risk of bias. Yellow circle with a '?' (question mark) symbol: Some concerns / Moderate risk of bias. Red circle with a '-' (minus) symbol: High risk / Serious risk of bias. Data Sources: [28, 29, 30] RoB 2.0: A revised Cochrane risk-of-bias tool for randomized trials by The Cochrane Collaboration, London, UK; ROBINS-I: Risk Of Bias In Non-randomized Studies - of Interventions by The Cochrane Collaboration, London, UK.

**Figure 3:**
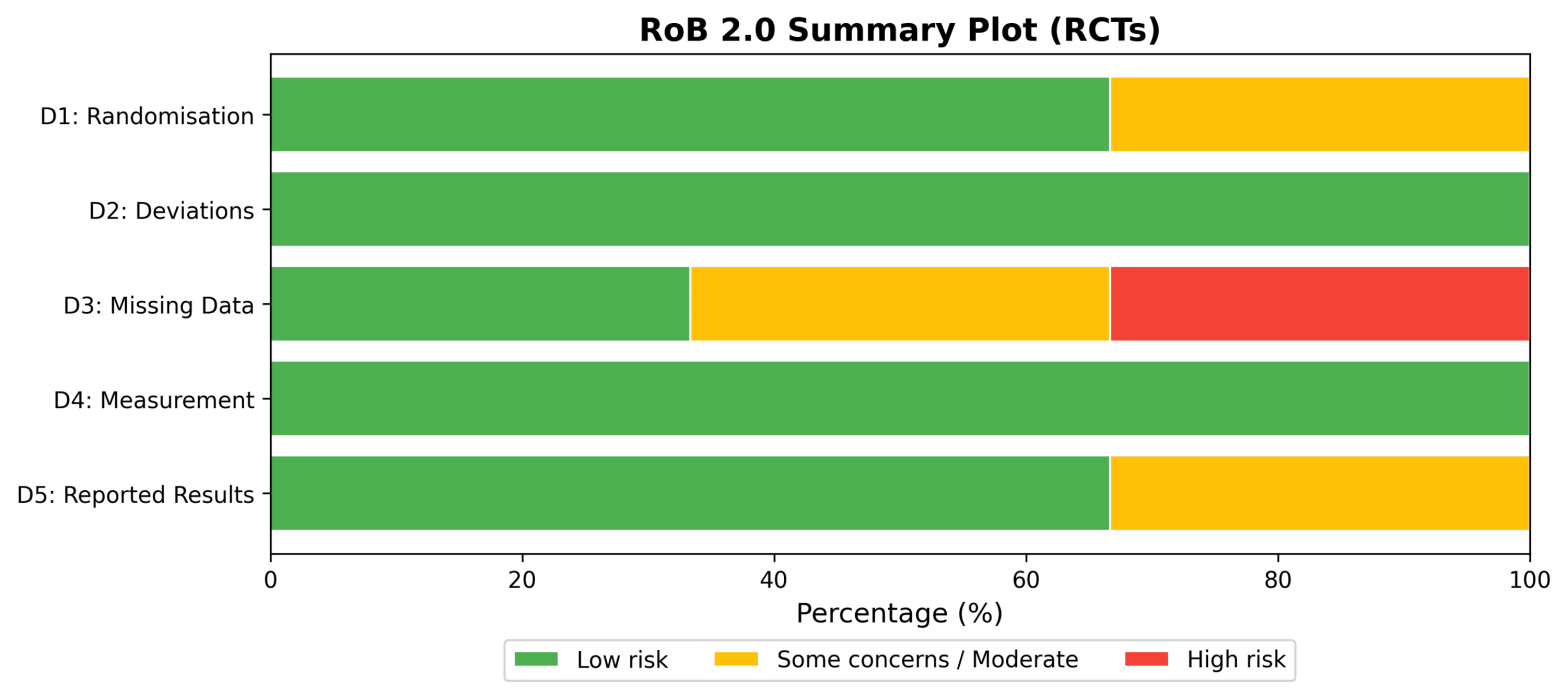
RoB 2.0 Summary Bar Plot Indicating the Proportion of RCTs With Low, Some Concerns, or High Risk of Bias. RoB 2.0: A revised Cochrane risk-of-bias tool for randomized trials by The Cochrane Collaboration, London, UK.

**Figure 4:**
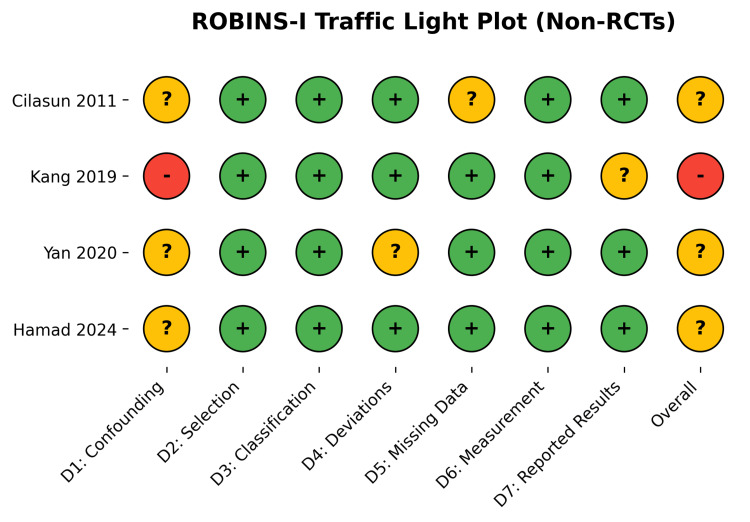
ROBINS-I Traffic Light Plot for Non-Randomized and Cohort Studies. The colors and symbols represent the domain-specific and overall risk-of-bias judgments according to the Cochrane RoB 2.0 and ROBINS-I tools. Green circle with a '+' (plus) symbol: Low risk of bias. Yellow circle with a '?' (question mark) symbol: Some concerns / Moderate risk of bias. Red circle with a '-' (minus) symbol: High risk / Serious risk of bias. Data Sources: [27, 31, 32, 33]. RoB 2.0: A revised Cochrane risk-of-bias tool for randomized trials by The Cochrane Collaboration, London, UK; ROBINS-I: Risk Of Bias In Non-Randomized Studies - of Interventions by the Cochrane Collaboration, London, UK.

**Figure 5:**
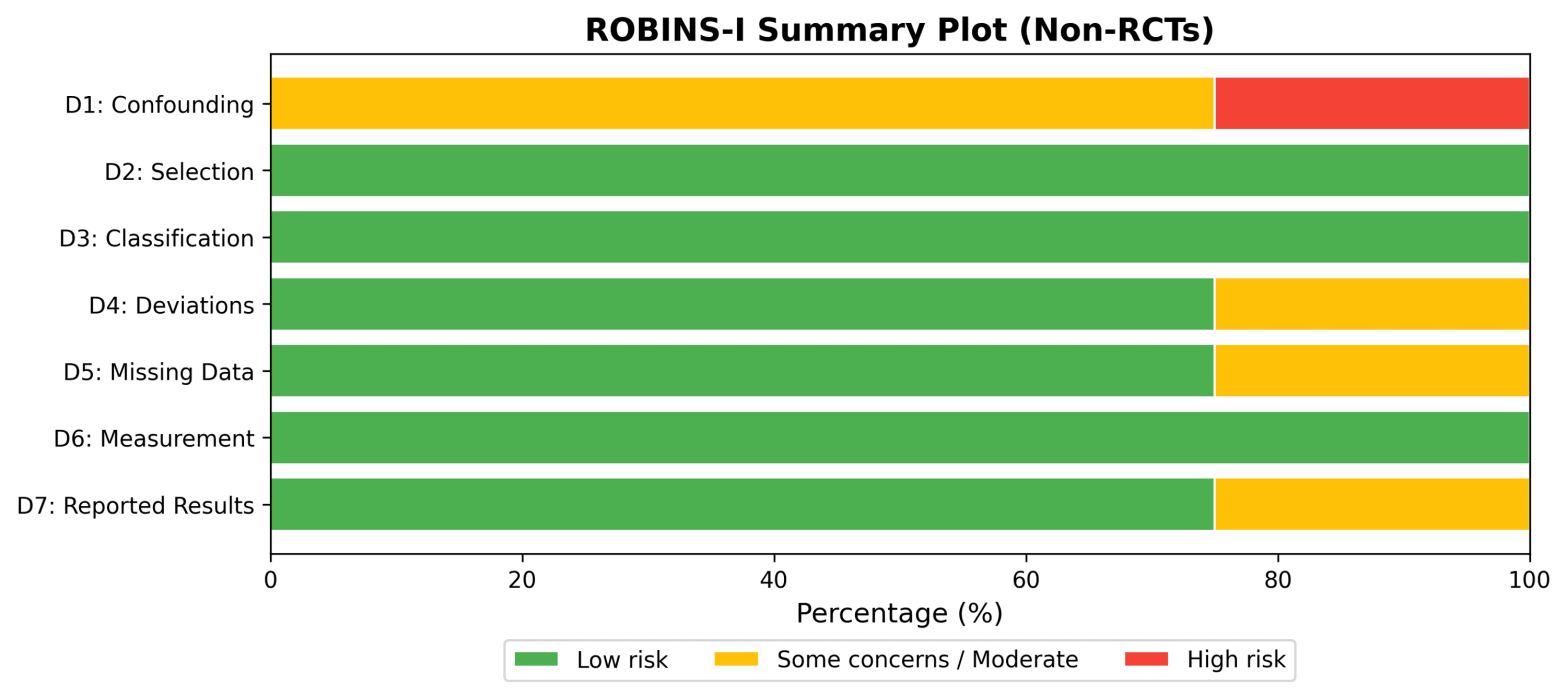
ROBINS-I Summary Bar Plot for Non-Randomized Studies. ROBINS-I: Risk Of Bias In Non-Randomized Studies - of Interventions by the Cochrane Collaboration, London, UK.

The methodological quality of the included RCTs [28,29,30] was assessed using the Cochrane RoB 2.0 tool. Overall, the RCTs demonstrated a low to moderate risk of bias, with some concerns primarily arising from deviations from intended interventions and missing outcome data in one study (Figures 2, 3) [30]. The non-randomized studies [26,27,31-33] were evaluated using the ROBINS-I tool. While selection, classification, and measurement domains were generally robust, confounding bias was the primary source of methodological limitation, resulting in an overall moderate to serious risk of bias across observational cohorts (Figures 4, 5).

Primary Outcome: Inferior Alveolar Nerve Injury (IANI)

Data for temporary and permanent IANI were pooled from six studies utilizing the Peto Odds Ratio method, which is mathematically optimized for rare events and zero-cell counts without utilizing artificial continuity corrections. Pang et al. [29] and Al-Ali et al. [26] were excluded from this specific contrast due to zero events in both study arms or missing data.

**Figure 6:**
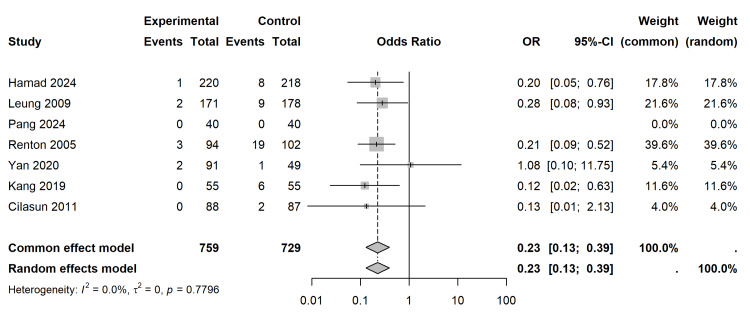
Primary Outcome Analysis: Inferior Alveolar Nerve Injury (IANI). Forest plot utilizing the Peto Odds Ratio method for rare events, demonstrating a significant reduction in IANI favouring coronectomy [27-33].

The pooled analysis demonstrated a profound and highly significant reduction in the incidence of IANI when coronectomy was performed compared to total extraction (Peto OR = 0.23; 95% CI (0.13, 0.39); p < 0.0001) (Figure 6). The studies were perfectly homogeneous for this outcome (I2 = 0.0%; τ2 = 0.00; Cochran’s Q = 2.48, p = 0.7796). Translating these relative findings into absolute clinical risk metrics, assuming a baseline Control Event Rate (CER) of 6.17% in the extraction group, the NNT is 21.2. A surgeon must substitute total extraction with coronectomy in approximately 22 high-risk patients to prevent one iatrogenic IAN injury.

Trial Sequential Analysis (TSA) for IANI

**Figure 7:**
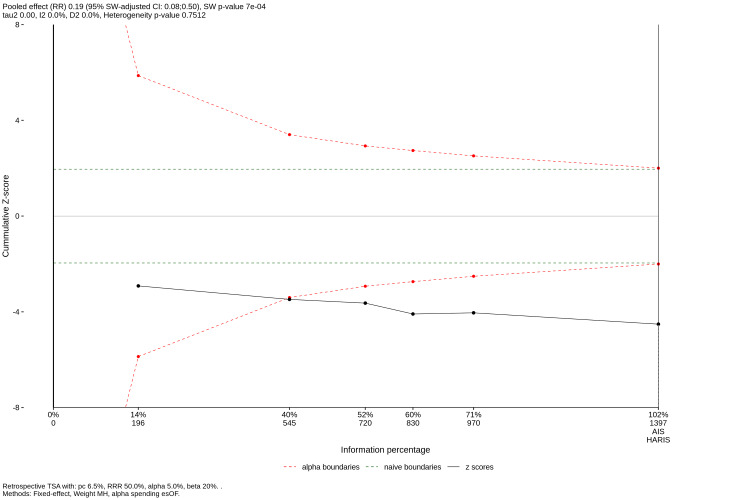
Trial Sequential Analysis (TSA) plot for IANI. The cumulative Z-curve (black line) crosses the trial sequential monitoring boundary (curved red dashed line) before the Required Information Size (RIS = 1,397), indicating conclusive evidence favouring coronectomy. RRR: Relative Risk Reduction; MH: Mantel-Haenszel (weighting method); esOF: error-spending O'Brien-Fleming (alpha-spending boundary); AIS: Actual Information Size; HARIS: Heterogeneity-Adjusted Required Information Size; SW: Satterthwaite-Welch (refers to the Satterthwaite-Welch approximation used for the adjusted confidence intervals and p-values).

To ascertain if the current body of evidence is robust against random errors and false-positive findings, a TSA was executed for the IANI outcome (Figure 7). Parameters were set a priori at an 80% power (β = 0.20), a 5% Type I error rate (α = 0.05), a CER of 6.5%, and a hypothesized Minimum Clinically Important Difference (Relative Risk Reduction) of 50%. The cumulative Z-curve (blue line) decisively crossed both the conventional significance boundary (Z = 1.96) and the O'Brien-Fleming trial sequential monitoring boundary before reaching the Required Information Size (RIS) of 1,397 patients, confirming that the evidence favouring coronectomy for IANI prevention is conclusive, and further RCTs evaluating this specific parameter are unlikely to alter the statistical outcome.

Secondary Outcomes

**Figure 8:**
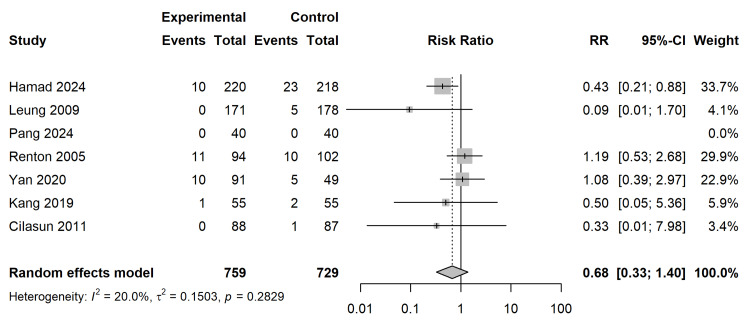
Secondary Outcome Analysis: Dry Socket. Forest plot demonstrating Risk Ratios (RR) utilizing REML variance estimation and HKSJ adjustment. The dark red bar represents the 95% Prediction Interval (PI) for future clinical settings [27-33]. REML: random-effects model; HKSJ: Hartung-Knapp-Sidik-Jonkman

Dry Socket (Alveolar Osteitis): Using a Random-Effects model with REML variance estimation and HKSJ adjustment, the analysis of 6 studies revealed a trend favouring coronectomy for the prevention of dry socket, though it did not achieve statistical significance (RR = 0.68; 95% CI (0.33, 1.40); p = 0.2246) (Figure 8). Statistical heterogeneity was low (I2 = 20.0%; τ2 = 0.1503; p = 0.2829). To assess future clinical applicability, a 95% Prediction Interval (PI) was generated, spanning [0.19, 2.42], indicating that in some future clinical settings, coronectomy might paradoxically increase the risk of dry socket.

**Figure 9:**
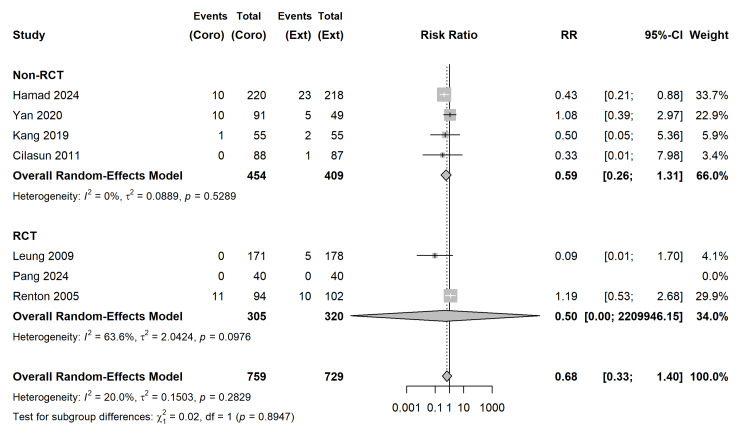
Subgroup Analysis Stratified by Study Design (RCT vs. Non-RCT). No significant interaction (p = 0.8947) [27-33]. Coro: Coronectomy; Ext: Total extraction

Subgroup analysis based on study design (RCT vs. Non-RCT) demonstrated no significant moderation effect on the risk of dry socket between subgroups (p = 0.8947) (Figure 9). Furthermore, meta-regression revealed that maximum follow-up duration (months) did not significantly act as a moderator for dry socket incidence (p = 0.4460).

**Figure 10:**
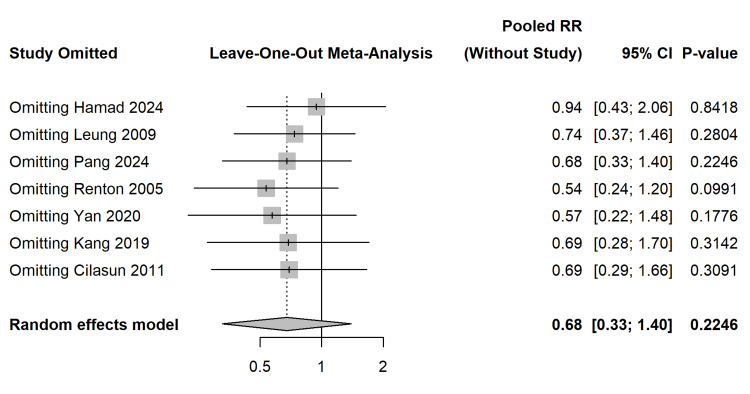
Leave-One-Out Sensitivity Analysis. Forest plot confirming the robustness of the pooled effect estimate regardless of the omission of any single study [27-33].

A leave-one-out sensitivity analysis confirmed the robustness of the pooled RR, as the sequential removal of individual studies did not shift the pooled estimate to statistical significance (RR range: 0.54 to 0.94) (Figure 10).

Postoperative Pain: Postoperative pain intensity, pooled via SMD, was reported in four studies [27,28,31,32]. The study by Al-Ali et al. [26] also reported pain via the Postoperative Symptom Severity (PoSSe) scale; however, it was excluded from the quantitative synthesis as the composite nature of the scale was incompatible with the Visual Analog Scale (VAS) metrics used in the other included trials. The HKSJ-adjusted random-effects model showed no statistically significant difference in pain between the coronectomy and extraction cohorts (SMD = -0.12; 95% CI (-0.59, 0.34); p = 0.4644). However, considerable statistical heterogeneity was observed (I2 = 79.6%; τ2 = 0.0642; p = 0.0021), stemming from the varied timing of pain assessments (e.g., Day 1 vs. Day 7) and different pain metrics utilized across studies.

Coronectomy-Specific Complications: Re-operation Rates

**Figure 11:**
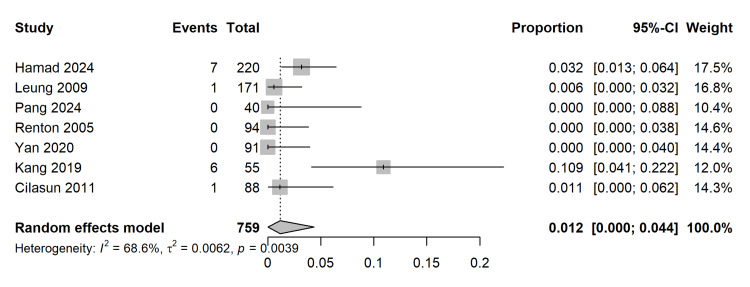
Single-Arm Proportional Meta-Analysis of Re-operation Rates. Forest plot showing the pooled incidence of re-operation following coronectomy, calculated using the Freeman-Tukey double arcsine transformation to stabilize variance in rare events [27-33].

A single-arm proportional meta-analysis was conducted for the seven studies reporting long-term outcomes following coronectomy. To stabilize variances and prevent confidence intervals outside the 0-1 bounds, the Freeman-Tukey double arcsine transformation was applied. The pooled incidence of requiring a secondary re-operation to remove retained/migrated roots was exceedingly low at 1.2% (Proportion = 0.012; 95% CI (0.000, 0.044)) (Figure 11). Moderate heterogeneity was present (I2 = 68.6%; p = 0.0039), reflecting variations in surgical technique (e.g., depth of root reduction below the crestal bone).

Publication Bias and Small-Study Effects

**Figure 12:**
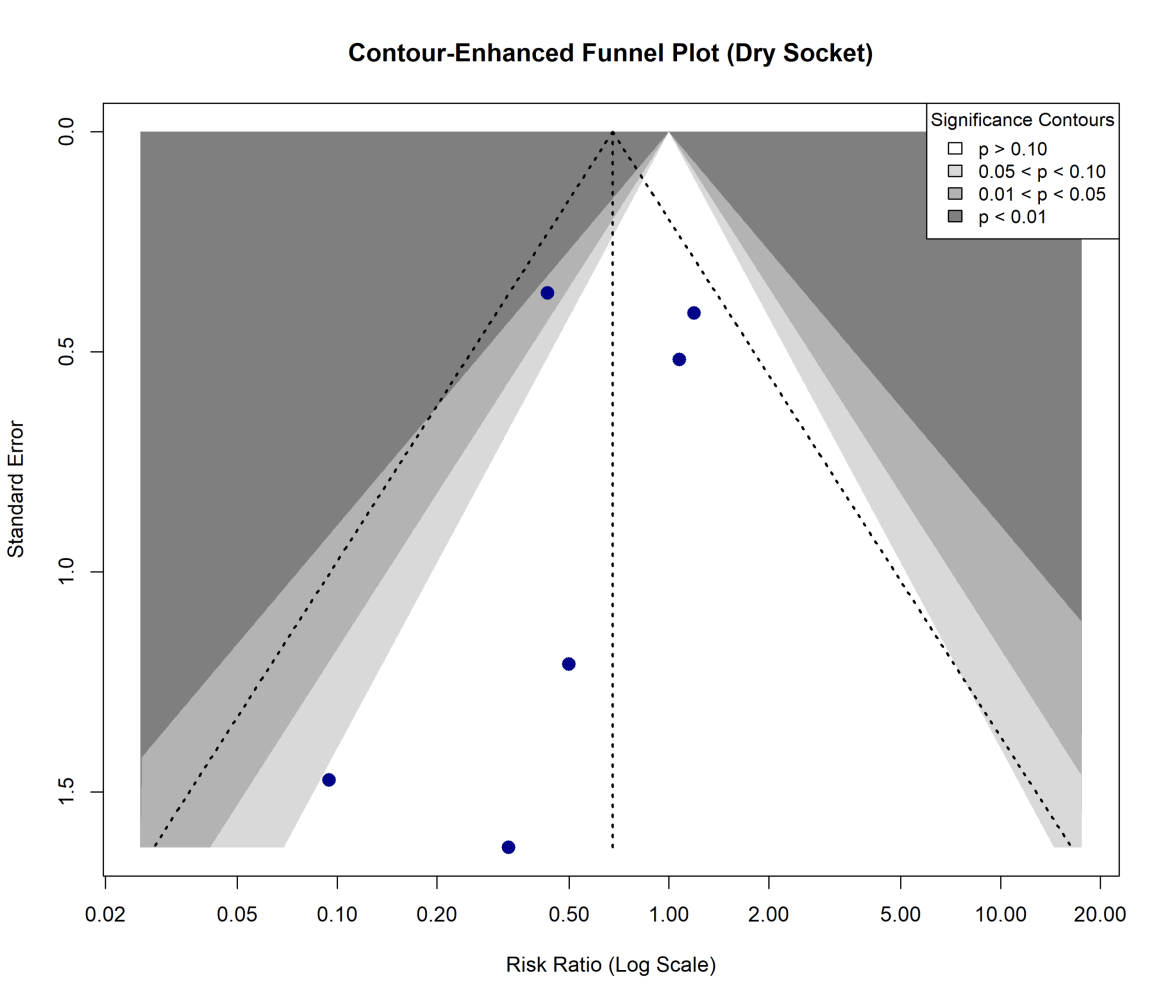
Contour-Enhanced Funnel Plot for Publication Bias. Visual assessment of small-study effects and publication bias for the dry socket outcome. Shaded contours represent levels of statistical significance (p < 0.10, 0.05, 0.01).

Potential reporting and dissemination biases were evaluated via a contour-enhanced funnel plot for the dry socket outcome (Figure 12). Visual inspection revealed mild asymmetry. While formal statistical testing for small-study effects was executed (Egger’s linear regression test and Begg’s rank correlation test), the limited number of eligible studies (k=6) constrains the statistical power of these tests. Therefore, the presence or absence of publication bias cannot be definitively confirmed based on these metrics alone.

Certainty of Evidence (GRADE Approach)

The overall strength and certainty of the evidence were appraised utilizing the GRADE framework (Table 2). The evidence supporting the reduction of IANI via coronectomy was graded as moderate; while the effect size was massive, it was downgraded one level due to imprecision (wide confidence intervals in several primary studies) and the inclusion of observational cohorts. Evidence regarding postoperative infection and dry socket was graded as moderate and low, respectively, driven by imprecision and risk of bias concerns within the non-randomized studies.

**Table 2:** GRADE Summary of Findings The ⨁ and ◯ symbols are standard visual indicators used in the GRADE framework to represent the overall certainty (quality) of the evidence. ⨁⨁⨁⨁ denotes High certainty; ⨁⨁⨁◯ denotes Moderate certainty; ⨁⨁◯◯ denotes Low certainty; ⨁◯◯◯ denotes Very low certainty. CI: Confidence Interval; NNT: Number Needed to Treat; OR: Odds Ratio; RR: Risk Ratio; TSA: Trial Sequential Analysis; GRADE: Grading of Recommendations Assessment, Development, and Evaluation.

Outcome	Anticipated Absolute Effects (per 1,000 patients)	Relative Effect (95% CI)	No. of Teeth (Studies)	Certainty of the Evidence (GRADE)	Comments
	Risk with Extraction	Risk Difference with Coronectomy			
Inferior Alveolar Nerve Injury (IANI) (Follow-up: 6–36 months)	77 per 1,000	63 fewer per 1,000 (from 69 fewer to 47 fewer)	Peto OR 0.23 [0.13, 0.39]	1,140 (6 studies)	⨁⨁⨁◯ MODERATE
Dry Socket (Alveolar Osteitis) (Follow-up: 1–4 weeks)	59 per 1,000	18 fewer per 1,000 (from 38 fewer to 21 more)	RR 0.68 [0.33, 1.40]	1,488 (7 studies)	⨁⨁◯◯ LOW
Postoperative Infection (Follow-up: 1 week – 24 months)	47 per 1,000	6 fewer per 1,000 (from 26 fewer to 35 more)	RR 0.87 [0.42, 1.79]	1,430 (8 studies)	⨁⨁◯◯ LOW
Re-operation (Root Retrieval) (Follow-up: 6–36 months)	N/A	12 per 1,000 (1.2% incidence)	Proportion: 0.012 [0.000, 0.044]	759 (7 studies)	⨁⨁◯◯ LOW

Discussion

The management of impacted mandibular third molars in close anatomical proximity to the IAN is a critical clinical challenge, where the imperative to treat pathology must be balanced against the risk of lifelong neurosensory morbidity. This systematic review and meta-analysis, encompassing eight studies and over 1,400 surgical cases, provides the most current and rigorous synthesis of evidence regarding the safety and efficacy of coronectomy versus total surgical extraction. The findings validate coronectomy as a superior neuroprotective modality, while also elucidating its specific risk profile regarding secondary complications.

Primary Outcome: Neuroprotection and Clinical Efficacy

The most pivotal finding of this study is the conclusive reduction in IAN injury rates associated with coronectomy. The pooled analysis of six comparative studies demonstrated a Peto Odds Ratio of 0.23 (p < 0.0001), translating to an approximate 77% relative risk reduction compared to total extraction. This aligns with and reinforces the conclusions of previous systematic reviews by Long et al. [34] and Cervera-Espert et al. [4], yet offers superior statistical robustness through the application of TSA. The TSA curve crossed the monitoring boundary for superiority, confirming that the current sample size is sufficient to declare coronectomy the safer alternative for high-risk cases without the need for further RCTs on this specific outcome. Clinically, the NNT of approximately 21 underscores the high efficiency of the procedure; for every 21 coronectomies performed in high-risk patients, one potentially permanent nerve injury is prevented. Given the medicolegal and quality-of-life implications of IAN injury, this metric provides a compelling argument for coronectomy as a primary treatment option for radiographically high-risk impactions to minimize neurological morbidity.

Secondary Morbidities: Infection and Dry Socket

Contrary to earlier concerns that retaining a root fragment might act as a nidus for infection, the meta-analysis found no statistically significant difference in the rates of postoperative infection (RR = 0.87) or dry socket (RR = 0.68) between coronectomy and total extraction, suggesting that the biological seal formed by the migration of the root remnant and subsequent bone healing is robust in the majority of cases [30,32]. The slightly lower (though non-significant) incidence of dry socket in the coronectomy group may be biologically plausible, attributable to the preservation of the alveolar socket architecture and a smaller clot volume requirement compared to the void left by a complete extraction [28,33]. However, the low certainty of evidence (GRADE) for these outcomes, driven by imprecision and study design limitations, mandates caution. Given the moderate-to-low certainty of evidence (GRADE), clinicians should counsel patients that while coronectomy does not eliminate standard surgical risks, it provides a safety profile comparable to conventional extraction regarding infection and dry socket.

Long-term Sequelae: Root Migration and Re-operation

A unique sequela of coronectomy is the migration of the retained root fragment. The analysis confirms that root migration is a frequent physiological response, occurring in most cases within the first 6-12 months before stabilizing [27,28,32]. While migration itself is rarely symptomatic, it can lead to root exposure or eruption into the oral cavity, requiring secondary retrieval. The pooled re-operation rate in our study was 1.2%, a figure that is clinically manageable and arguably an acceptable trade-off for neuroprotection. When re-operation is required, the root has typically migrated away from the mandibular canal, rendering the second procedure significantly safer than the initial high-risk extraction would have been [30,32]. However, the heterogeneity in re-operation rates (I2 = 68%) suggests variability in surgical technique (e.g., inadequate reduction of the root below the crestal bone) or follow-up protocols across centers, which highlights the necessity for strict adherence to the standardized protocol of reducing the root at least 3 mm below the alveolar crest to facilitate optimal bone coverage [5,26].

Methodological Strengths and Limitations

This review improves upon prior meta-analyses by incorporating recent high-quality data [27,29], utilizing robust statistical models (HKSJ adjustment, Peto method for rare events), and employing TSA to control for Type I errors. The stratification of evidence quality using GRADE provides a transparent assessment of certainty for clinicians.

The inclusion of non-randomized cohort studies introduces potential selection bias, as evidenced by the moderate risk of bias in the observational subset. Furthermore, the variability in imaging modalities (Panoramic vs. CBCT) across studies may influence the preoperative classification of high-risk cases, although our subgroup analyses did not find this to be a significant moderator. The lack of uniform reporting standards for pain and quality of life metrics prevented a more granular meta-analysis of patient-reported outcomes (PROMs), an area identified as a key gap in the current literature [26].

## Conclusions

Current evidence supports coronectomy as a safe and effective alternative to total extraction for mandibular third molars with a high risk of inferior alveolar nerve injury. The procedure significantly reduces the incidence of nerve damage without increasing the risk of dry socket or postoperative infection. While root migration is common, the need for re-operation is rare and typically low risk. While the certainty of evidence for secondary outcomes remains low to moderate, the conclusive neuroprotective benefits suggest that coronectomy should be considered a primary treatment option for high-risk impactions in clinical practice guidelines. Future research should prioritize the standardization of PROMs to better capture the patient experience and long-term quality of life.
